# Assessing the development of mental fatigue during simulated flights with concurrent EEG-fNIRS measurement

**DOI:** 10.1038/s41598-023-31264-w

**Published:** 2023-03-23

**Authors:** Anneke Hamann, Nils Carstengerdes

**Affiliations:** Deutsches Zentrum für Luft- und Raumfahrt e.V. (DLR), Institut für Flugführung, Lilienthalplatz 7, 38108 Braunschweig, Germany

**Keywords:** Psychology, Aerospace engineering, Cognitive neuroscience

## Abstract

Mental fatigue (MF) can impair pilots’ performance and reactions to unforeseen events and is therefore an important concept within aviation. The physiological measurement of MF, especially with EEG and, in recent years, fNIRS, has gained much attention. However, a systematic investigation and comparison of the measurements is seldomly done. We induced MF via time on task during a 90-min simulated flight task and collected concurrent EEG-fNIRS, performance and self-report data from 31 participants. While their subjective MF increased linearly, the participants were able to keep their performance stable over the course of the experiment. EEG data showed an early increase and levelling in parietal alpha power and a slower, but steady increase in frontal theta power. No consistent trend could be observed in the fNIRS data. Thus, more research on fNIRS is needed to understand its possibilities and limits for MF assessment, and a combination with EEG is advisable to compare and validate results. Until then, EEG remains the better choice for continuous MF assessment in cockpit applications because of its high sensitivity to a transition from alert to fatigued, even before performance is impaired.

## Introduction

Flying an aircraft is a complex task that requires visual and auditory attention, cognitive and motor control, and working memory capacity^1^. Even during nominal cruise flights where the pilots’ tasks mainly consist of monitoring the aircraft systems and interacting with air traffic control, a low to medium but constant task load is put upon them for a prolonged period of time. Still, the pilots are expected to perform well at all times and to quickly adapt to unforeseen changes^2^. However, prolonged task execution can lead to mental fatigue (MF)^3^. MF is associated with impaired action monitoring, response preparation and error correction^4,5^. It can thus influence the pilots’ decision making^6^, and impair their ability to react to changes and to strategically modify their behaviour. Therefore, the assessment of MF is of great interest in aviation research, especially with respect to safety, accident prevention and the development of adaptive assistance systems.

The exact definitions and terms used to describe MF vary considerably across the literature^7,8^. One of the most prominent definitions is Grandjean’s (1979). He describes MF as a state of reduced alertness, verging towards but not within the sleepy spectrum, that is produced by prolonged task execution and/or highly demanding tasks and that can be counteracted by taking breaks^3^. MF is also seen as an adaptive strategy of the brain that indicates an imbalance between spent resources and the level of goal attainment, thereby reflecting both depletion of cognitive resources and motivational aspects^9^. MF is sometimes used interchangeably (and somewhat imprecisely) with sleepiness, drowsiness or tiredness^10^, shows substantial overlap with the concepts of vigilance decrement^11^ and sustained attention^12^ or is simply referred to as time on task (ToT)^13^. Furthermore, in some experiments MF is induced by a combination of sleep deprivation and ToT^14–16^, thereby further diluting the boundaries between fatigued and sleepy. This multitude of definitions and terms makes MF a concept that is difficult to pinpoint, with empirical findings that are hard to compare across studies. For the present work, we follow Grandjean’s (1979) definition of MF^3^, i.e. acute, non-pathological mental fatigue induced by demanding or prolonged tasks, like flying an aircraft.

Physiological measures are of increasing interest for MF detection because of their objectiveness, high temporal resolution and ability to detect MF-related changes as early as 45 min into task execution^17^. While electroencephalography (EEG) is among the most widely used assessment methods, functional near-infrared spectroscopy (fNIRS) has recently gained much attention in aviation^8^. In the EEG signal, MF usually elicits an increase in parietal alpha power^18–21^ that is associated with a shift from attention to the default mode which suppresses external stimulus processing^22–24^. This alpha increase is usually accompanied by an increase in frontal theta power^18,19,21^ which typically reflects cognitive control, i.e. control of task execution, memory function and error processing^23,25^. Increasing theta activity is thought to reflect higher effort to meet the increasing cognitive demands of the prolonged task^19,26^. Moreover, higher power in slow frequency bands (i.e. theta, alpha) is associated with lower performance^23,27^, giving further evidence that the accumulation of MF may deteriorate performance over time. Increasing parietal beta power is sometimes also associated with growing MF^18,20^, but these effects are found less consistently^19^. In fNIRS, the findings are somewhat heterogeneous. MF is usually associated with increasing frontal cortical activation, expressed as increasing levels of oxyhaemoglobin (HbO) and decreasing levels of deoxyhaemoglobin (HbR)^12,20,28,29^. However, some studies also report a decrease in activation after prolonged task execution^30^ and decreasing connectivity across frontal and parietal areas with increasing MF^31^. Finally, there are studies that could not find consistent changes over time or in only one channel^32,33^.

While there is a large body of MF-related research on EEG and fNIRS in the context of driving^20,28–31,33^, only few studies have focused specifically on pilots^21,34,35^. In addition, tasks, durations, montages and analysis strategies vary considerably between studies. Specifically, the fNIRS-based MF detection on pilots was done using only classification algorithms^34,35^ and no information on the direction of changes or time course of the relevant measures is available yet. In addition, fNIRS results are seldom compared to more established measures such as EEG to validate the results. These aspects pose a challenge for the application of empirical findings to the cockpit. A precise, valid, reliable assessment of pilots’ cognitive capacity is vital to providing support, especially when it comes to adaptive assistance systems. Previous research has shown that an interaction of MF and other factors such as mental workload (MWL) decreases the ability to correctly identify each separate factor^36^. As long as the physiological assessment of pilots’ cognitive capacity lacks systematic investigation, comparable methodology and rigorous control of confounding factors, this will limit the validity and reliability of results. In turn, this will negatively impact the development and quality of adaptive assistance systems.

We want to contribute to the systematic investigation of MF and MWL. In our previous research, we successfully detected different levels of MWL with concurrent EEG-fNIRS measurement in the flight simulator while explicitly controlling for confounding effects of MF^37^. The current paper is the second step in this systematic investigation and presents an essential expansion of our previous research. This time, our aim was to induce MF while controlling for confounding effects of MWL, and to find valid physiological measures to assess it. We induced MF via ToT using a 90-min simulated flight task derived from our previous study^37^. It had a low, constant task difficulty to control for influences of MWL. To ensure comparability with our previous study, we used a similar experimental paradigm and the same concurrent EEG-fNIRS montage, flight simulator, tasks and material, and analysis strategy. We hypothesized that increasing ToT would lead to: increasing self-reported MF; decreasing performance in both sub-tasks of the simulated flight task; increasing EEG frontal theta power and parietal alpha and beta power; increasing frontal cortical activation in fNIRS (i.e. decreasing HbR, increasing HbO); and no changes in self-reported MWL (whereas an increase would indicate a confounding effect). This study has been preregistered at AsPredicted.org.

## Results

### Subjective data

**Figure 1:**
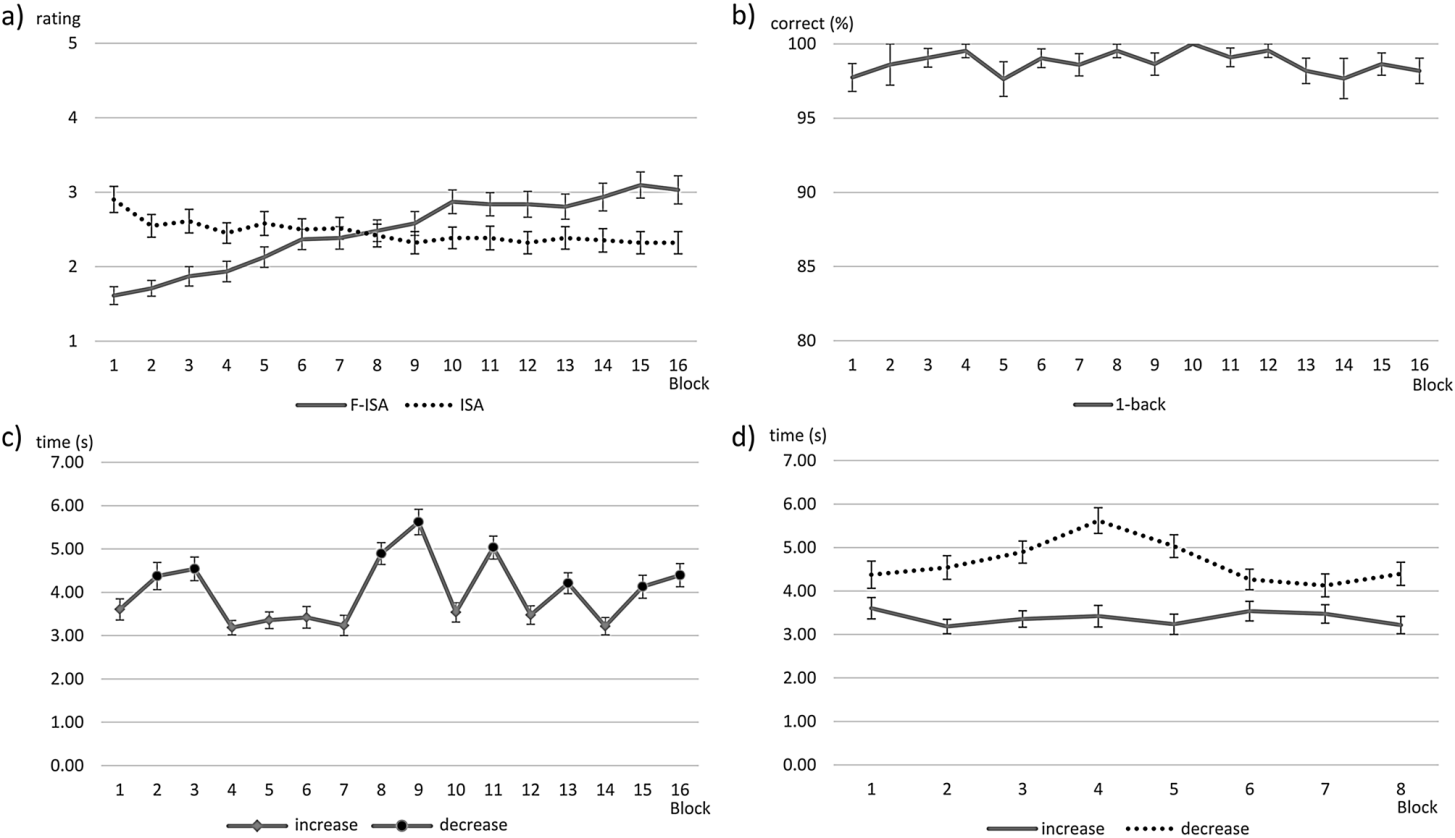
Mean subjective and performance measures across blocks. Error bars indicate *SE*. (**a**) F-ISA and ISA ratings. (**b**) 1-back accuracy (%). (**c**) Monitoring reaction time (s) including direction of the altitude deviation. (**d**) Monitoring reaction times (s) split by direction.

Subjective data showed a high sensitivity to MF changes with increasing ToT, see Table 1. Self-reported MF increased substantially over time. Mean KSS ratings rose significantly from 2.81 (“alert”; *SD* = 1.38) before the experiment to 5.39 (“neither alert nor sleepy”; *SD* = 1.94) after the experiment, *t*(30) = − 7.66, *p* < 0.001. Mean F-ISA ratings increased by 1.42 points between block 1 and 16, from 1.61 (“low”) to 3.03 (“medium/relaxed wakeful”), see Fig. 1a). The ANOVA showed a significant effect of ToT on F-ISA, *F*(3.69, 110.54) = 26.02, *p* < 0.001, η^2^_p_ = 0.46 with a linear trend, *F*(1, 30) = 54.24, *p* < 0.001, η^2^_p_ = 0.64. Subsequent t-tests showed significant differences between all tested blocks (i.e. start, ¼, ½, ¾, end of the experiment), all *p*s ≤ 0.083, one-tailed, see Table 2.

**Table 1:** Overview of different MF measures and their sensitivity to increasing time on task (i.e. blocks).

	Self-report	EEG—frontal theta activity			EEG—parietal alpha activity			fNIRS—channel AF7–AFF5h	
Comparison of blocks	F-ISA	Fz	F3	F4	Pz	P3	P4	HbR	HbO
1 vs. 4	**✔**				**✔**	**✔**	**✔**		
1 vs. 8	**✔**				**✔**	**✔**	**✔**		
1 vs. 12	**✔**		**✔**	**✔**	**✔**	**✔**	**✔**	**✔**	
1 vs. 16	**✔**	**✔**	**✔**	**✔**	**✔**	**✔**	**✔**		
4 vs. 8	**✔**				**✔**	**✔**	**✔**		
4 vs. 12	**✔**		**✔**		**✔**	**✔**	**✔**		
4 vs. 16	**✔**	**✔**			**✔**	**✔**	**✔**		
8 vs. 12	**✔**		**✔**						
8 vs. 16	**✔**		**✔**	**✔**					**✔**
12 vs. 16	**✔**								

Significant differences between blocks marked with **✔** (for self-report and EEG one-tailed, i.e. p < 0.1; for fNIRS two-tailed, i.e. *p* < 0.05). Measures without significant differences are omitted.

**Table 2:** Paired t-tests for F-ISA and ISA, significant comparisons only (F-ISA one-tailed, ISA two-tailed).

Measure	Comparison		Mean difference (*SE*)	*p*-value
F-ISA	1 vs	4	− 0.32 (0.10)	0.002
		8	− 0.87 (0.14)	< 0.001
		12	− 1.23 (0.17)	< 0.001
		16	− 1.42 (0.20)	< 0.001
	4 vs	8	− 0.55 (0.15)	0.001
		12	− 0.90 (0.15)	< 0.001
		16	− 1.10 (0.19)	< 0.001
	8 vs	12	− 0.36 (0.14)	0.014
		16	− 0.55 (0.17)	0.002
	12 vs	16	− 0.19 (0.11)	0.083
ISA	1 vs	4	0.45 (0.12)	0.001
		8	0.48 (0.15)	0.004
		12	0.58 (0.18)	0.004

Alpha levels are Bonferroni-Holm corrected.

Self-reported MWL (ISA) did not increase with ToT, but rather decreased substantially within the first quarter of the experiment. The mean ratings decreased from 2.90 (“comfortable”, *SD* = 0.98) in block 1 to 2.45 (“relaxed”, *SD* = 0.77) in block 4 and to 2.32 (“relaxed”, *SD* = 0.83) in block 16, see Fig. 1a). The ANOVA showed a significant effect, *F*(4.38, 131.41) = 2.53, *p* = 0.039, η^2^_p_ = 0.08 with a weak linear trend, *F*(1, 30) = 4.22, *p* = 0.049, η^2^_p_ = 0.12. Subsequent t-tests showed significant differences between block 1 and blocks 4, 8 and 12, respectively (all *p*s ≤ 0.004, two-tailed), see Table 2 and Supplement Table S1.

### Performance data

Performance did not decrease steadily with increasing ToT. 1-back performance showed a ceiling effect (> 97% correct reactions in all blocks), see Fig. 1b), and the ANOVA did not show a significant effect of ToT, *p* > 0.05. Reaction times in the monitoring task varied significantly with time, *F*(7.31, 219.37) = 13.77, *p* < 0.001, η^2^_p_ = 0.32, see Fig. 1c). An exploratory 2 (direction) × 8 (block) repeated-measures ANOVA revealed a significant main effect of the direction of the altitude change, *F*(1, 30) = 45.74, *p* < 0.001, η^2^_p_ = 0.60, of block, *F*(7, 210) = 3.72, *p* = 0.001, η^2^_p_ = 0.11, and the interaction, *F*(7, 210) = 4.34, *p* < 0.001, η^2^_p_ = 0.13. Reactions to an altitude increase were generally faster than reactions to a decrease, see Fig. 1d). Further exploratory t-tests between the 1., 4. and 8. block per direction revealed significant changes over time only in reactions to an altitude decrease. The reactions were slower in the fourth block compared to the first and last, respectively, *p*s ≤ 0.001.

### EEG data

**Figure 2:**
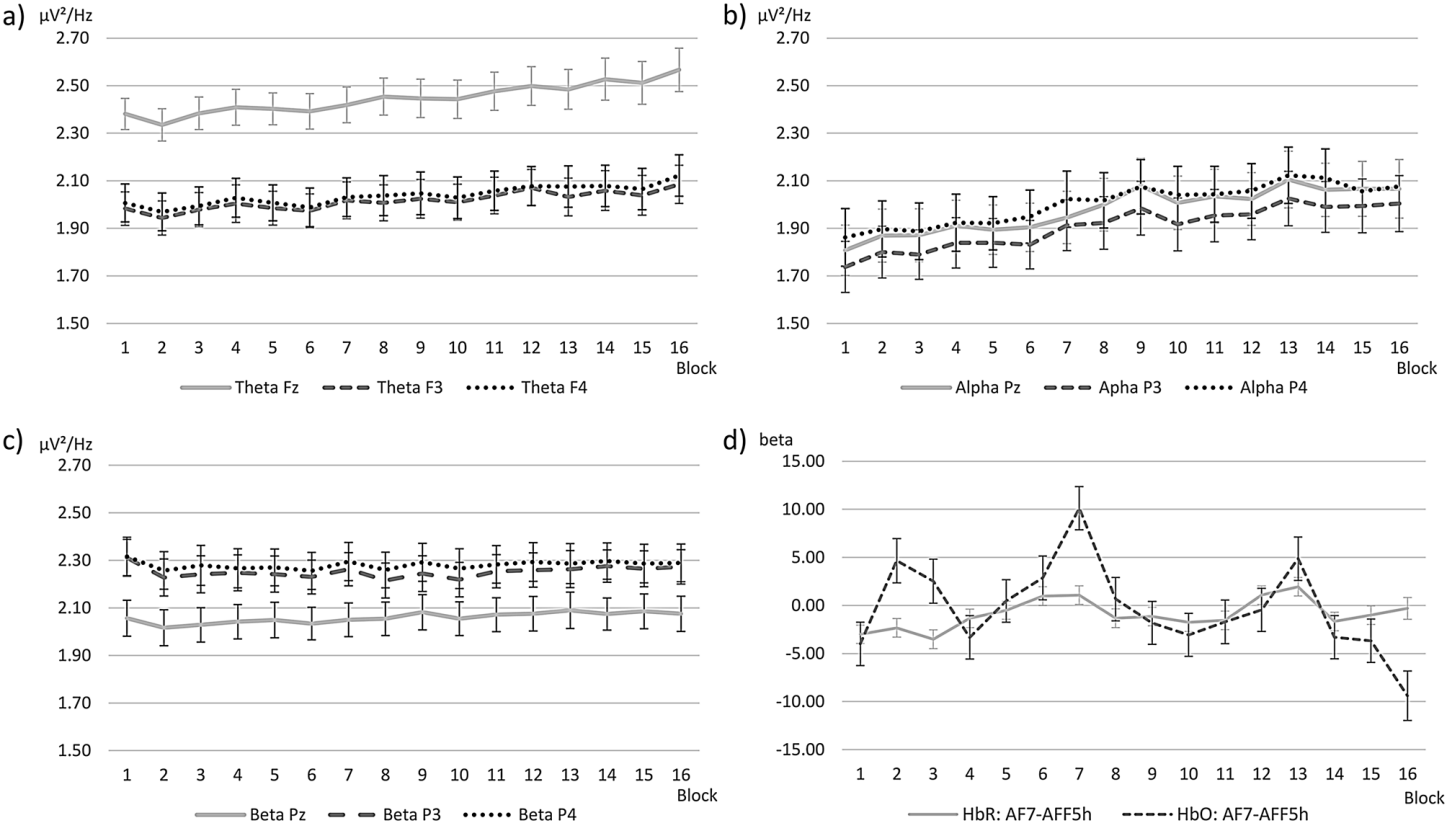
Physiological measures across blocks. Error bars indicate *SE*. EEG measures are mean Power Spectral Density (ln-transformed): (**a**) Frontal theta; (**b**) Parietal alpha; (**c**) Parietal beta. (**d**) fNIRS beta values for the channel with significant differences and a linear trend in HbR and HbO.

EEG data were sensitive to MF changes with increasing ToT, and different frequency bands showed different abilities to discriminate the changes over time, see Table 1. Frontal theta activity increased linearly with ToT, see Fig. 2a). Separate ANOVAs revealed significant differences for all three frontal sites, electrode Fz, *F*(2.46, 73.69) = 7.56, *p* < 0.001, η^2^_p_ = 0.20 with a linear trend, *F*(1, 30) = 11.40, *p* = 0.002, η^2^_p_ = 0.28, electrode F3, *F*(4.56, 136.92) = 4.97, *p* = 0.001, η^2^_p_ = 0.14 with a linear trend, *F*(1, 30) = 10.03, *p* = 0.004, η^2^_p_ = 0.25, and electrode F4, *F*(5.11, 153.18) = 5.95, *p* < 0.001, η^2^_p_ = 0.17 with a linear trend, *F*(1, 30) = 14.00, *p* = 0.001, η^2^_p_ = 0.32. Subsequent t-tests revealed significant differences only between the early (1, 4, 8) and the late blocks (12, 16), indicating a gradual theta activity increase that only reached significance towards the end of the experiment, see Table 3 and Supplement Table S2.

**Table 3:** Paired t-tests for EEG Theta and Alpha activity, significant comparisons only (one-tailed).

Measure	Comparison		Mean difference (*SE*)	*p*-value
Theta Fz	1 vs	16	− 0.19 (0.06)	0.004
	4 vs	16	− 0.16 (0.05)	0.004
Theta F3	1 vs	12	− 0.09 (0.03)	0.007
		16	− 0.10 (0.04)	0.014
	4 vs	12	− 0.07 (0.03)	0.016
	8 vs	12	− 0.06 (0.02)	0.008
		16	− 0.08 (0.03)	0.008
Theta F4	1 vs	12	− 0.07 (0.03)	0.010
		16	− 0.12 (0.04)	0.003
	8 vs	16	− 0.08 (0.03)	0.003
Alpha Pz	1 vs	4	− 0.10 (0.03)	0.001
		8	− 0.19 (0.03)	< 0.001
		12	− 0.22 (0.04)	< 0.001
		16	− 0.26 (0.04)	< 0.001
	4 vs	8	− 0.09 (0.02)	< 0.001
		12	− 0.11 (0.03)	0.001
		16	− 0.16 (0.04)	< 0.001
Alpha P3	1 vs	4	− 0.10 (0.04)	0.008
		8	− 0.18 (0.04)	< 0.001
		12	− 0.22 (0.04)	< 0.001
		16	− 0.27 (0.05)	< 0.001
	4 vs	8	− 0.08 (0.03)	0.012
		12	− 0.12 (0.03)	0.001
		16	− 0.17 (0.04)	< 0.001
Alpha P4	1 vs	4	− 0.06 (0.03)	0.023
		8	− 0.16 (0.03)	< 0.001
		12	− 0.20 (0.03)	< 0.001
		16	− 0.21 (0.04)	< 0.001
	4 vs	8	− 0.09 (0.03)	0.004
		12	− 0.13 (0.03)	< 0.001
		16	− 0.15 (0.03)	< 0.001

Alpha levels are Bonferroni-Holm corrected.

Parietal alpha activity also increased linearly with ToT, see Fig. 2b). The ANOVAs revealed significant differences for all three parietal sites, electrode Pz, *F*(7.16, 214.76) = 13.94, *p* < 0.001, η^2^_p_ = 0.32 with a linear trend, *F*(1, 30) = 68.82, *p* < 0.001, η^2^_p_ = 0.70, electrode P3, *F*(7.68, 230.30) = 10.98, *p* < 0.001, η^2^_p_ = 0.27 with a linear trend, *F*(1, 30) = 11.40, *p* = 0.002, η^2^_p_ = 0.62, and electrode P4, *F*(8.23, 246.91) = 11.26, *p* < 0.001, η^2^_p_ = 0.27 with a linear trend, *F*(1, 30) = 11.40, *p* = 0.002, η^2^_p_ = 0.65. Subsequent t-tests revealed significant differences between the early blocks 1 and 4 and all respective later blocks (first half of the experiment), but no significant differences between blocks 8, 12 and 16 (second half of the experiment), indicating a stronger increase of parietal alpha activity within the first half of the experiment, see Table 3 and Supplement Table S2.

For parietal beta activity, a significant increase over time was only found at the central parietal site Pz, see Fig. 2c). The ANOVA revealed a significant difference for electrode Pz, *F*(8.45, 253.57) = 3.72, *p* < 0.001, η^2^_p_ = 0.11 with a linear trend, *F*(1, 30) = 16.79, *p* < 0.001, η^2^_p_ = 0.36. Subsequent t-tests did not yield significant results. The ANOVAs for electrodes P3 and P4 did not reach significance.

### fNIRS data

Two channels showed significant and opposing linear trends in HbR and HbO, indicating decreasing cortical activation over time: AF7-AFF5h on the left hemisphere (HbR: *t* = 3.12, *p* = 0.013; HbO: *t* = − 2.90, *p* = 0.019), and AFF6h-AF8 in the mirrored location on the right hemisphere (HbR: *t* = 3.26, *p* = 0.010; HbO: *t* = − 3.61, *p* = 0.006). There was a significant positive trend in four more channels in HbR (all *t*s(479) ≥ 2.54, all *p*s ≤ 0.035), and in three more channels in HbO (all *t*s(479) ≥ 2.65, all *p*s ≤ 0.030). See Supplement Table  S3 for complete statistical values and Supplement Fig. S1 for visualisation. Discrimination between blocks was only possible for block 1 vs. later blocks, and block 16 vs. earlier blocks in both HbR and HbO (with one exception: block 8 vs. 12 in HbO), see Table 4. AF7-AFF5h was the only channel with a linear and opposing trend in HbR and HbO and discrimination ability between at least two blocks (see Table 1 and Fig. 2d). Further investigation showed significant differences between the activity in the blocks and baseline (i.e. before task onset) mainly in the first and last block, see Table 4.

**Table 4:** Haemodynamic results for significant t-tests per channel in HbR and HbO.

Type	Block	Comparison	Channel	β (*SE*)	*t*	*p*-value
HbR	1 vs	Baseline	FFC4h–FFC6h	− 5.01 (1.33)	− 3.78	0.007
			AF7–AFF5h	− 2.99 (0.95)	− 3.14	0.034
		4	FFC4h–FFC6h	5.56 (1.63)	3.41	0.014
		8	FFC4h–FFC6h	5.48 (1.63)	3.36	0.016
		12	FFC4h–FFC6h	5.99 (1.63)	3.67	0.010
			AF7–AFF5h	4.08 (1.11)	3.66	0.010
		16	FFC4h–FFC6h	6.69 (1.77)	3.79	0.007
	4 vs	16	FFC4h–FFC2h	6.12 (1.89)	3.24	0.023
	8 vs	16	FFC4h–FFC2h	5.89 (1.89)	3.12	0.030
	12 vs	16	FFC4h–FFC2h	5.81 (1.87)	3.10	0.031
	16 vs	Baseline	FFC4h–FFC2h	5.68 (1.59)	3.56	0.013
HbO	1 vs	Baseline	FFC5h-AFF5h	16.35 (3.35)	4.88	0.000
			FFC5h-FFC3h	− 10.36 (2.46)	− 4.21	0.002
		4	FFC5h-AFF5h	− 27.24 (4.66)	− 5.84	< 0.001
		8	FFC5h-AFF5h	− 16.84 (4.68)	− 3.60	0.010
		12	FFC5h-AFF5h	− 30.92 (4.68)	− 6.61	< 0.001
		16	AFF6h-AF8	− 13.11 (3.74)	− 3.51	0.012
			AFF3h-AFF5h	15.04 (4.70)	3.20	0.025
			FCC3h-FFC3h	15.16 (4.29)	3.54	0.012
	4 vs	Baseline	FFC5h-AFF5h	− 10.89 (3.54)	− 3.08	0.036
		16	AFF3h-AFF5h	23.12 (4.68)	4.94	< 0.001
			FFC5h-FFC3h	− 12.27 (3.59)	− 3.42	0.014
			FCC3h-FFC3h	21.17 (4.25)	4.98	< 0.001
	8 vs	Baseline	FFC5h-FFC3h	− 9.78 (2.39)	− 4.09	0.002
		12	FFC5h-AFF5h	− 14.09 (4.74)	− 2.97	0.043
		16	AF7-AFF5h	− 10.04 (3.26)	− 3.08	0.031
			AFF3h-AFF5h	20.23 (4.65)	4.35	0.001
			FCC3h-FFC3h	19.59 (4.19)	4.67	< 0.001
	12 vs	Baseline	FFC5h-AFF5h	− 14.57 (3.44)	− 4.23	0.002
		16	AFF3h-AFF5h	24.41 (4–64)	5.26	< 0.001
			FFC5h-AFF5h	16.25 (5.17)	3.14	0.029
			FFC5h-FFC3h	− 12.33 (3.55)	− 3.47	0.013
			FCC3h-FFC3h	15.01 (4.18)	3.59	0.010
	16 vs	Baseline	AF7-AFF5h	− 9.38 (2.58)	− 3.64	0.011
			AFF3h-AFF5h	20.99 (3.64)	5.76	< 0.001
			FFC5h-FFC3h	− 19.12 (2.84)	− 6.74	< 0.001
			FCC3h-FFC3h	14.07 (3.32)	4.24	0.002

Comparisons against baseline (before task onset) and against other blocks are presented. *df* = 479. FDR-corrected *p*-values^38^.

## Discussion

In this study we aimed at inducing MF with a simulated 90-min flight task. We controlled for confounding effects of MWL by keeping the task difficulty constant, and used self-report, task performance and physiological data as means of assessing MF across time.

Our experiment successfully induced MF across time without any confounding effects of MWL. Subjective MF ratings (F-ISA) increased substantially and linearly over time, from low to medium fatigued. KSS ratings increased similarly, from alert to a state between alert and sleepy. The increase is comparable to previous studies of similar duration^39,40^. Self-reported MWL did not increase with ToT. A slight decrease from “comfortable” to “relaxed” was visible within the first quarter of the experiment, indicative of a learning effect with a small effect size.

Neither performance measure showed the expected decrease over time. The 1-back performance exhibited a ceiling effect (> 97% accuracy throughout all blocks). Monitoring reaction times varied with ToT, but without a clear trend. Exploratory analyses revealed a substantial difference between the directions of the altitude deviation: Reactions to an altitude decrease were generally slower than reactions to an increase. This unexpected finding is due to the behaviour of the flight simulator and incorporated flight physics. The simulator performs altitude decreases slightly slower and less steadily than increases, and therefore the reaction threshold for decreases is reached later. This makes reaction times between the two directions incomparable, but should not have interacted with ToT. This was, however, the case. Only the slower reactions seem to be affected by ToT and showed a substantial increase towards the middle of the experiment (ca. 45–50 min after task onset). It is unlikely that this effect is due to motivational aspects because the participants were not told how much time had passed or how many blocks were yet to come, and changes in motivation should have affected both the slow and the fast reactions. This could not be observed, even though there were blocks in the opposite direction immediately before and after this peak. However, we could not find differences between the altitude deviation directions in any other measure and thus conclude that this did not interfere with the overall accumulation of MF.

The EEG data provide clear evidence for a change from an alert to a fatigued state. Parietal alpha activity exhibited the expected linear increase over time^18,26,40,41^ on all three electrodes of interest. This increase was strongest within the first 45 min and seemed to level out towards the end of the experiment. In frontal theta activity, the expected linear increase over time^18,26,36^ could be observed. However, substantial differences in theta activity could only be found between the late and early blocks and less consistently across the three electrodes of interest. Parietal beta activity increased slightly over time, but could not be used to differentiate between blocks. This is not surprising, as there is evidence both for^18^ and against^19^ changes in beta power with increasing MF. In sum, we found a clear pattern of MF-associated changes in EEG band power that is consistent with the literature. The time course of EEG activity during our simulated flight task can be described as a fast and substantial alpha increase at parietal regions very early on (0–45 min). Alpha then stagnates while frontal theta increases slower but steadily across the whole 90 min. A similar time course of early alpha increase and levelling, accompanied by a steady theta increase over time has been shown in earlier work^19,26^.

The obtained fNIRS results do not show such a clear and consistent picture of MF development. Following previous results^12,29,30^, we expected increasing cortical activation (i.e. decreasing HbR and increasing HbO) with growing MF. We found linear temporal trends for some of the channels, but only two showed the fNIRS-typical pattern of opposite direction changes in HbR and HbO. Contrary to our expectations, HbR increased and HbO decreased over time. Differentiation between the blocks and from baseline activity (i.e. before task onset) was possible mainly for the first and last block, and the results varied considerably across channels and between HbR and HbO. It is therefore likely that the linear trends across time are mainly driven by the activity in the beginning and at the end of the experiment, and not by a gradual change.

In HbR, we observed substantial decreases during the first block where MF could not have accumulated yet. Apart from MF, decreases in HbR are also elicited by higher task difficulty^37,42^. The reduced HbR in the beginning of the experiment therefore likely mirrors the learning effect found in subjective ratings. The increase in HbR in the last block corresponds with findings of reduced cortical activation after three hours of simulated driving^30^. However, we could not observe a consistent HbR reduction across our 90-min experiment that would indicate a gradual accumulation of MF. It is therefore unlikely that the increase in the last block is due to MF. It is similarly unlikely that the changes in the last block were related to sudden motivational changes or task disengagement because the participants were not informed that the experiment was nearly finished. In order to establish if this increase was the beginning of a trend or a singular event, a longer experiment would have been necessary. In HbO, we found increases as well as decreases in the same blocks and in neighbouring channels. In other words, the direction of the changes was very location-specific and did not follow a general pattern, similar to previous research^32,33^. There is evidence that HbO is more susceptible to contamination by systemic noise than HbR^43,44^. It has been argued that it is therefore less useful in driving simulations that involve a lot of movement^42^. This could also be the case for our simulated flight task, and could explain why HbO changes were even less consistent than HbR changes.

The demands of our experiment may not have been high enough to elicit the typically found changes in cortical oxygenation. The typically observed increased frontal activation over time is thought to counteract the detrimental effects of MF on performance^29^. Due to the low task difficulty (as seen in low MWL ratings and the ceiling effect in 1-back performance), the participants may not have needed to engage further resources to keep their performance on an acceptable level. This idea aligns with our EEG results: The increase in frontal theta power is also seen as a compensation for MF^19,26^. In our experiment, theta power increased only slowly and reached significance later than the increasing parietal alpha power. In an experiment with higher task difficulty or an even longer duration, the participants might have needed to recruit more cognitive resources to keep their performance stable. As a result, frontal activation would have been higher both in fNIRS and EEG. In light of these considerations on frontal cortical activation, future studies should also consider including parietal fNIRS measurement, because there is evidence that fNIRS can detect changes in attention and vigilance in parietal areas^45^, similar to parietal EEG activity. As it is, frontal fNIRS measurement seems less sensitive to MF changes than EEG in tasks with constant but low cognitive demand.

It is noteworthy that if we had only compared the fNIRS measurements of the first and last block, as is widely done (at the beginning and end of the experiment^12^ or in intervals of one hour^28,32,33^), we would have attributed the changes we found to MF. The lacking consistency of any trend in any direction, however, makes this interpretation implausible. We would therefore like to stress the need to collect and analyze fNIRS data continuously to gain insight into the time course of MF-related changes instead of mere pre-post comparisons. Like researchers before us^46^, we would also like to highlight the importance of comparability of fNIRS methodology and the replication of findings in order to get a better understanding of MF-related changes in fNIRS and their dependence on factors like measured cortical area, task length or difficulty, and analysis strategies.

In sum, EEG activity proved most sensitive to MF over the course of 90 min. It showed substantial increases in frontal theta and parietal alpha power even though performance was not yet impaired. In contrast, frontal cortical oxygenation did not change systematically overt time. From our findings we draw two main inferences for aviation research. First, in order to establish if fNIRS has the potential for MF detection over time, more research is needed and a combination and comparison with an established measure like EEG is advisable to draw valid conclusions. Second, it is important to monitor pilots’ MF development with physiological measures because an increase in MF can go unnoticed and without performance deterioration in a task with constant and low difficulty such as a nominal cruise flight. A sudden change or unforeseen situation might then overexert the fatigued pilot’s cognitive resources and lead to poor reactions and decisions. An early detection of MF development could prevent such situations, and EEG is still the most viable measurement for this application.

## Methods

### Sample

In order to minimize confounding effects of experience and age, we recruited a student sample. Participants were recruited via mailing lists of the Technical University Braunschweig and the German Aerospace Center (DLR) Braunschweig. Inclusion criteria were: native German speakers, currently enrolled at a university, right-handedness, normal or corrected-to-normal vision, normal hearing, no pre-existing neurological conditions, no flying experience, no pilot’s licence or radio telephony certificate and an initial score of six or less on the Karolinska Sleepiness Scale (KSS^47^, German version^48^), i.e. not within the “sleepy” spectrum. One participant was excluded due to not being enrolled at a university. Therefore, the final sample comprised 31 participants (20 male, 11 female) between 19 and 33 years (*M* = 24.1, *SD* = 3.4). The participants were asked to follow their normal sleep and caffeine habits prior to the experiment. They provided written informed consent and received 30 € for participation. The study was approved by the ethics commission of the German Psychological Society (DGPs) and conducted in accordance with the declaration of Helsinki.

### Flight simulation

**Figure 3:**
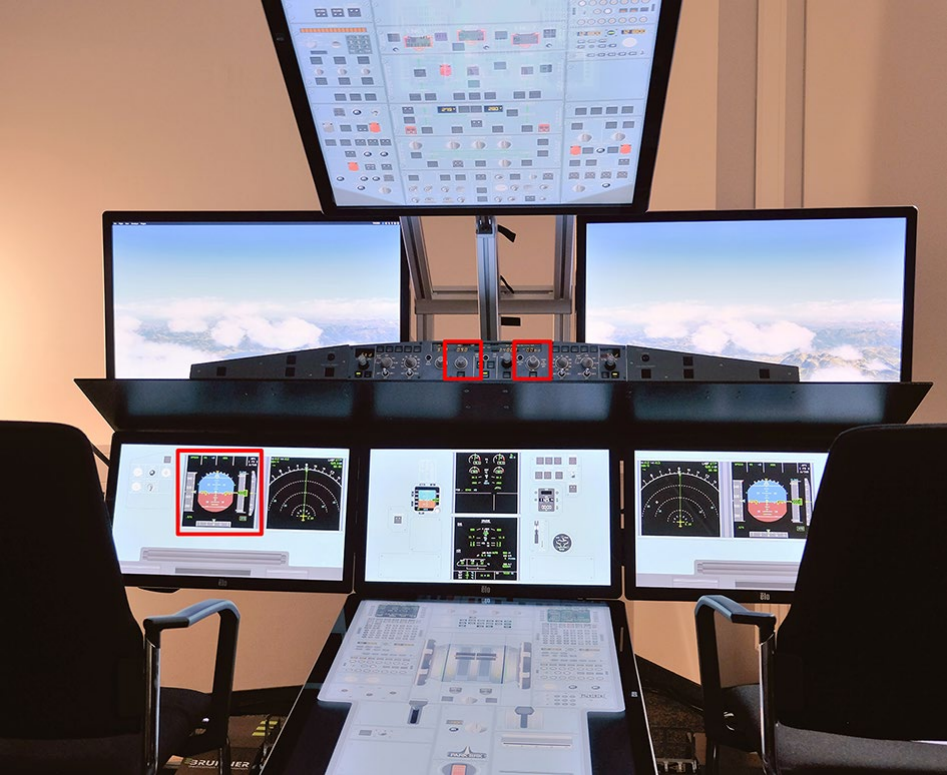
The iSim A321 cockpit. Primary flight display (left) and hardware panel with heading dial (centre left) and vertical speed dial (centre right) for required inputs are marked in red.

The simulated flight task was designed to be easy to learn without prior flight experience, but required cognitive functions similar to a realistic nominal cruise flight. It consisted of two parallel tasks described in the following sections. For the task, an A321 cockpit was simulated in the iSim, a flight simulator based on X-Plane 11 (Laminar Research, Columbia, SC, USA) and situated at the Institute of Flight Guidance at DLR Braunschweig. The participants sat on the left side, monitored the primary flight display and made the required inputs via the heading dial and vertical speed dial (Fig. 3).

### Experimental tasks and material

The experiment had a one-factorial (ToT) within-subject design. It consisted of 16 blocks of approx. three minutes each. In every block, the participants had to execute two parallel tasks of constant difficulty, followed by subjective ratings, and a two-minute break. The total time of the experiment was approx. 90 min.

#### Monitoring task

Participants monitored the altitude of the aircraft (initially 20,000 ft in each block). If they noticed a deviation of 40 ft or more, they had to readjust the altitude as fast as possible using the vertical speed dial. Exactly one deviation per block was triggered by the experimenter. Onset and direction (50% up, 50% down) were randomized across blocks, but fixed within a block, so that every participant experienced the same altitude direction change at the same time in the same block.

#### Adapted 1-back task

In order to induce constant low working memory load, we used the 1-back condition of the adapted n-back task described in our previous study^37^. The task was programmed in PsychoPy3 v2020.1^49^. Auditory heading commands, i.e. required course corrections, served as stimuli. The participants heard a sequence of heading commands and had to follow them in line with the 1-back instruction: They had to set the heading of the aircraft to the heading one prior to the one just heard by using the heading dial. For this task, 16 lists were generated, each consisting of eight heading commands and eight randomized inter-stimulus intervals (*M* = 22 s, *SD* = 3 s) to prevent confounding of the fNIRS data with Mayer waves^50,51^. The order of the lists was randomized for each participant.

#### Subjective measures

General level of sleepiness was assessed upon arrival (for exclusion purposes only), and right before and after the experiment with the German version of the KSS^47,48^ on a 1–9 scale (“extremely alert” to “very sleepy”). MF was assessed with the F-ISA^52^ on a 1–5 scale (“very low/alert” to “very high/fatigued”). MWL was assessed with the ISA^53,54^ on a 1–5 scale (“underutilized” to “excessive workload”). Both F-ISA and ISA ratings were given verbally after each block.

### Procedure

Each experimental session started at 9:30 am to control for circadian variance effects, and lasted approx. 3.5 h. Lighting conditions of the room, and volume of the iSim and stimuli were kept constant. Upon arrival, the participants completed a demographic questionnaire, the initial KSS assessment and received instructions. Then, concurrent EEG and fNIRS recordings were prepared and calibrated and the participants practiced three blocks of the two parallel tasks. They all achieved the minimal criterion of 60% correct reactions in at least one of the blocks. To avoid learning effects, the initial heading was set to 090 during practice and to 270 during the main experiment. Feedback was only given during practice. Thereafter, the participants completed a second KSS assessment and moved on to the main experiment. Finally, they filled in the third KSS assessment, were thanked and compensated.

### Physiological data recording

**Figure 4:**
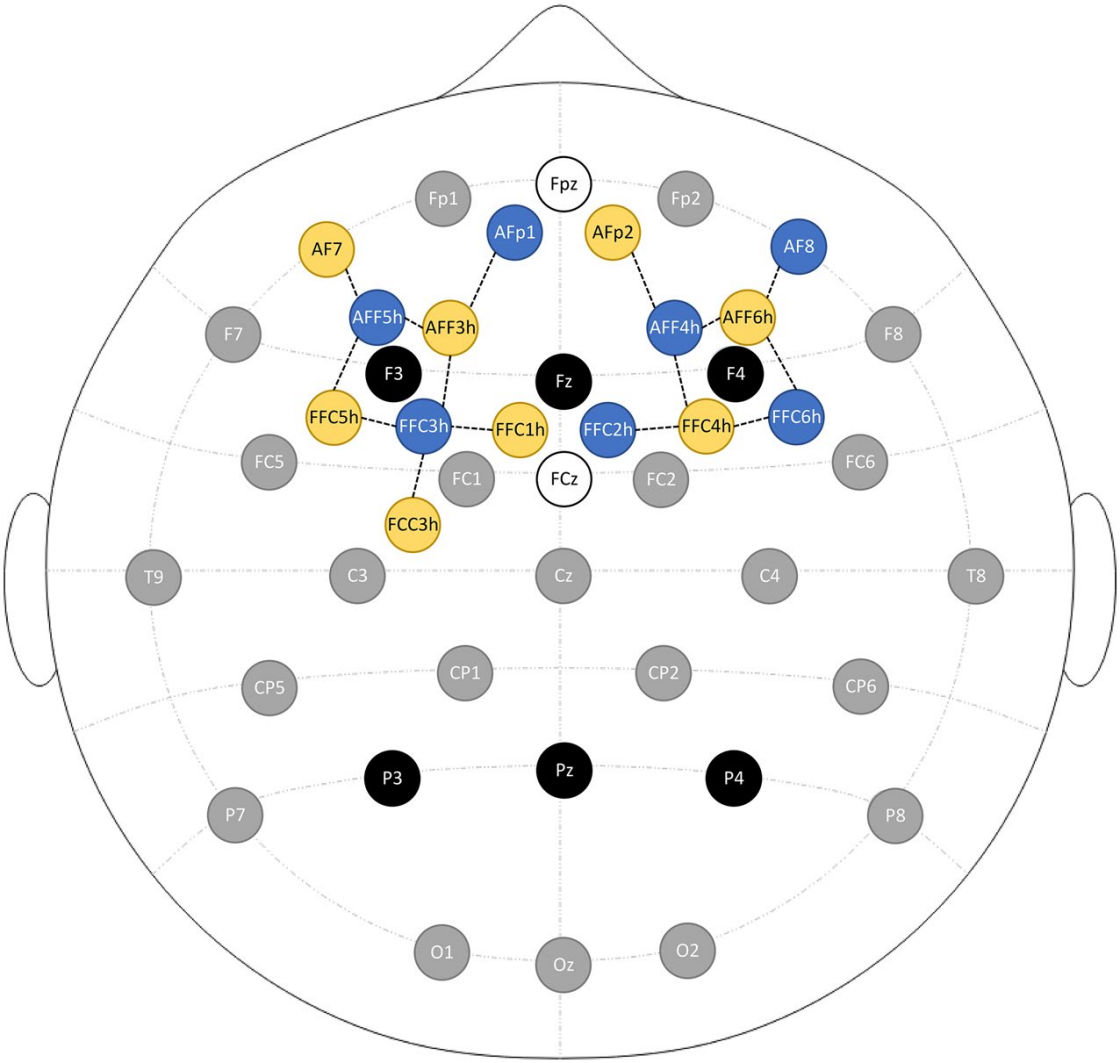
Combined EEG-fNIRS montage. Black = electrode used in analysis, grey = electrode not used in analysis, white = reference and ground electrodes, blue = fNIRS detector, yellow = fNIRS source incl. short distance channel, dashed lines = fNIRS channel.

EEG signals were recorded from 28 Ag/AgCl active electrodes at 500 Hz with a LiveAmp-32 in BrainVision Recorder 1.24 (Brain Products GmbH, Gilching, Germany). The electrodes were positioned according to the 10–20 system with online reference at FCz. fNIRS optodes were positioned in between to form 15 channels (see Fig. 4). Recording was performed at 10 Hz with a NIRSport2 with eight sources, seven detectors and eight short-distance channels, in the software Aurora 2021.9 (NIRx Medical Technologies LLC, Glen Head, NY, USA). Further details on the montage design can be found in our previous study^37^.

### Data analysis

Statistical analyses were conducted in SPSS 26 (IBM Corp., Armonk, NY, USA) and MATLAB R2020a (The MathWorks Inc, Nattick, MA, USA). Missing values (1.1% of the performance and subjective data, 0.2% in EEG data, none in fNIRS data) were replaced by mean substitution, i.e. replacement by the mean of the non-missing cases of the respective variable where necessary. If not specified otherwise, a one-factorial (ToT, i.e. 16 blocks) repeated measures analysis of variance (ANOVA) with polynomial contrasts was conducted for each measure. In case of violation of the sphericity assumption, Greenhouse–Geisser corrected values are reported. The polynomial contrasts were used to determine the trend of the data. For each analysis, only the function with the highest fit (determined by η^2^_p_) is reported. Each ANOVA with a significant outcome was followed up with Bonferroni-Holm corrected paired t-tests for the blocks 1, 4, 8, 12 and 16 (start, ¼, ½, ¾ and end of the experiment). The t-tests were on-tailed for directional hypotheses, and two-tailed for non-directional hypotheses and exploratory analyses.

#### Subjective data

F-ISA and ISA values were analysed with ANOVAs as described above. KSS values before and after the experiment were compared using a one-tailed paired t-test.

#### Performance data

For the 1-back task, performance (in percent) was computed for each block as the ratio of correct to all responses. If more than one response per heading command was registered, this was counted as uncertainty and therefore the overall response to this heading command was considered incorrect. An ANOVA was conducted as described above. For the monitoring task, reaction time (in seconds) to the altitude deviation per block was used as a performance measure. Seven far-outliers according to Tukey’s fences (3 × interquartile range) were identified and replaced by mean substitution. An ANOVA was conducted as described above. An exploratory 2 (direction) × 8 (block) repeated-measures ANOVA was used to test if the direction of the altitude change had an impact on reaction times, followed by Bonferroni-Holm corrected paired t-tests for the 1., 4. and 8. block per direction.

#### Physiological data

EEG data was pre-processed in BrainVision Analyzer 2.2 (Brain Products GmbH, Gilching, Germany). The data was down-sampled to 256 Hz, re-referenced to average and bandpass-filtered between 0.5 and 40 Hz using a 4th order IIR filter with an additional 50 Hz notch filter to remove remaining line noise from the unshielded simulator. Motion artefacts were removed after visual inspection aided by the semi-automatic artefact rejection procedure in the aforementioned software, and an independent component analysis was performed for ocular correction. The data was then divided in blocks beginning with the first reaction per block, and further segmented into epochs of 2 s with 0.5 s overlap. Power Spectral Density was computed using Fast Fourier Transformation with a Hanning window with 10% overlap. The average per block was exported as raw sum (µV^2^/Hz) for the bands and electrodes of interest: For the theta (4–8 Hz) band at electrodes Fz, F3 and F4; and for the alpha (8–13 Hz) and beta (13–30 Hz) bands at electrodes Pz, P3 and P4. The data was ln-transformed to account for skewness. An initially planned multivariate analysis of variance (MANOVA) per frequency band could not be computed due to insufficient residual degrees of freedom. Instead, only the planned subsequent separate ANOVAs for each frequency band and electrode were computed as described above. A Bonferroni-Holm correction was applied to the three ANOVAs per band.fNIRS data pre-processing and analysis was done using the NIRS Brain AnalyzIR toolbox for MATLAB^55^. The data was down-sampled to 4 Hz, converted from raw voltage to optical density, then to relative concentration of oxygenated and deoxygenated haemoglobin using the modified Beer-Lambert Law^56^. The data was divided into 16 blocks beginning with the first reaction per block and entered into a two-level mixed-effects general linear model (GLM). On the subject level, for each channel and block a regression coefficients beta was computed using the gamma hemodynamic response function^57^. These beta values indicate the magnitude of hemodynamic changes compared to baseline (before task onset). Serial autocorrelation, motion artefacts and physiological confounds were corrected using pre-whitening (AR-IRLS^58^) and by including the short-distance channels as regressors^59^. On the group level, the blocks were included as fixed effects and subjects as random effects into the model. A linear contrast was computed on the beta values across all blocks to check for linear trends with ToT. T-tests were computed between blocks 1, 4, 8, 12 and 16. For these channels, comparisons against baseline were used to gain deeper insight into the origin of the effects. All *p*-values were corrected using the false-discovery rate (FDR)^38^.

## Supplementary Information

Supplementary Information.

## Data Availability

The pre-registration, data and scripts for analysis are publicly available: Pre-registration: https://aspredicted.org/fh77h.pdf; Data: http://dx.doi.org/10.23668/psycharchives.8306; Scripts: http://dx.doi.org/10.23668/psycharchives.8307.
