# Supplementary material for: Assessing the development of mental fatigue during simulated flights with concurrent EEG-fNIRS measurement
Source: Sci Rep. 2023 Mar 23;13:4738. doi: 10.1038/s41598-023-31264-w (PMC10036528; doi:10.1038/s41598-023-31264-w)
Supplement: Supplementary file 1 — Supplementary Information. [file 41598_2023_31264_MOESM1_ESM.pdf]

Supplements

Tables

Measure	Comparison		Mean difference (*SE*)	*p*-value
F-ISA	1 vs.	4	-0.32 (0.10)	.002*
		8	-0.87 (0.14)	< .001*
		12	-1.23 (0.17)	< .001*
		16	-1.42 (0.20)	< .001*
	4 vs.	8	-0.55 (0.15)	.001*
		12	-0.90 (0.15)	< .001*
		16	-1.10 (0.19)	< .001*
	8 vs.	12	-0.36 (0.14)	.014*
		16	-0.55 (0.17)	.002*
	12 vs.	16	-0.19 (0.11)	.083*
ISA	1 vs.	4	0.45 (0.12)	.001*
		8	0.48 (0.15)	.004*
		12	0.58 (0.18)	.004*
		16	0.58 (0.21)	.010
	4 vs.	8	0.03 (0.13)	.801
		12	0.13 (0.13)	.325
		16	0.13 (0.15)	.403
	8 vs.	12	0.10 (0.09)	.264
		16	0.10 (0.13)	.448
	12 vs.	16	0.00 (0.09)	> .999

Table S1. Exhaustive results of the paired t-tests for F-ISA and ISA including non-significant comparisons. Alpha levels are Bonferroni-Holm corrected. Significant comparisons (F-ISA one-tailed, ISA two-tailed) marked *.

Measure	Comparison		Mean difference (*SE*)	*p*-value
Theta Fz	1 vs.	4	-0.03 (0.02)	.252
		8	-0.07 (0.03)	.024
		12	-0.12 (0.05)	.016
		16	-0.19 (0.06)	.004*
	4 vs.	8	-0.05 (0.02)	.013
		12	-0.09 (0.03)	.014
		16	-0.16 (0.05)	.004*
	8 vs.	12	-0.04 (0.03)	.142
		16	-0.11 (0.04)	.015
	12 vs.	16	-0.07 (0.03)	.013
Theta F3	1 vs.	4	-0.02 (0.02)	.392
		8	-0.02 (0.02)	.271
		12	-0.09 (0.03)	.007*
		16	-0.10 (0.04)	.014*
	4 vs.	8	0.00 (0.02)	.890
		12	-0.07 (0.03)	.016*
		16	-0.08 (0.04)	.059
	8 vs.	12	-0.06 (0.02)	.008*
		16	-0.08 (0.03)	.008*
	12 vs.	16	-0.01 (0.03)	.608

[Table S2 continued]

Theta F4	1 vs.	4	-0.02 (0.02)	.257
		8	-0.03 (0.02)	.102
		12	-0.07 (0.03)	.010*
		16	-0.12 (0.04)	.003*
	4 vs.	8	-0.01 (0.02)	.587
		12	-0.05 (0.02)	.039
		16	-0.09 (0.04)	.017
	8 vs.	12	-0.04 (0.02)	.035
		16	-0.08 (0.03)	.003*
	12 vs.	16	-0.04 (0.02)	.076
Alpha Pz	1 vs.	4	-0.10 (0.03)	.001*
		8	-0.19 (0.03)	< .001*
		12	-0.22 (0.04)	< .001*
		16	-0.26 (0.04)	< .001*
	4 vs.	8	-0.09 (0.02)	< .001*
		12	-0.11 (0.03)	.001*
		16	-0.16 (0.04)	< .001*
	8 vs.	12	-0.02 (0.03)	.380
		16	-0.07 (0.03)	.038
	12 vs.	16	-0.04 (0.03)	.224
Alpha P3	1 vs.	4	-0.10 (0.04)	.008*
		8	-0.18 (0.04)	< .001*
		12	-0.22 (0.04)	< .001*
		16	-0.27 (0.05)	< .001*
	4 vs.	8	-0.08 (0.03)	.012*
		12	-0.12 (0.03)	.001*
		16	-0.17 (0.04)	< .001*
	8 vs.	12	-0.04 (0.03)	.258
		16	-0.08 (0.04)	.039
	12 vs.	16	-0.04 (0.03)	.162
Alpha P4	1 vs.	4	-0.06 (0.03)	.023*
		8	-0.16 (0.03)	< .001*
		12	-0.20 (0.03)	< .001*
		16	-0.21 (0.04)	< .001*
	4 vs.	8	-0.09 (0.03)	.004*
		12	-0.13 (0.03)	< .001*
		16	-0.15 (0.03)	< .001*
	8 vs.	12	-0.04 (0.03)	.253
		16	-0.06 (0.04)	.142
	12 vs.	16	-0.02 (0.03)	.574

Table S2. Exhaustive results of the paired t-tests for EEG Theta and Alpha activity including non-significant comparisons. Alpha levels are Bonferroni-Holm corrected. Significant comparisons (one-tailed) marked *.

Type	Channel	β (*SE*)	*t*	*p*-value
HbR	AFF6h-AF8	119.52 (36.62)	3.26	.010
	FFC4h-FFC2h	121.86 (47.91)	2.54	.035
	FFC4h-FFC6h	152.01 (45.58)	3.34	.010
	FFC1h-FFC3h	125.57 (45.15)	2.78	.024
	AF7-AFF5h	97.57 (31.29)	3.12	.013
	FFC5h-AFF5h	131.51 (50.00)	2.63	.030
HbO	AFF6h-AF8	-348.34 (96.62)	-3.61	.006
	FFC4h-AFF4h	374.77 (126.66)	2.96	.018
	FFC1h-FFC3h	241.79 (91.25)	2.65	.030
	AF7-AFF5h	-250.84 (86.58)	-2.90	.019
	FCC3h-FFC3h	434.19 (109.47)	3.97	.003

Table S3. Haemodynamic results for significant linear trends per channel in HbR and HbO. *df* = 479. FDR-corrected *p*-values.

Figures

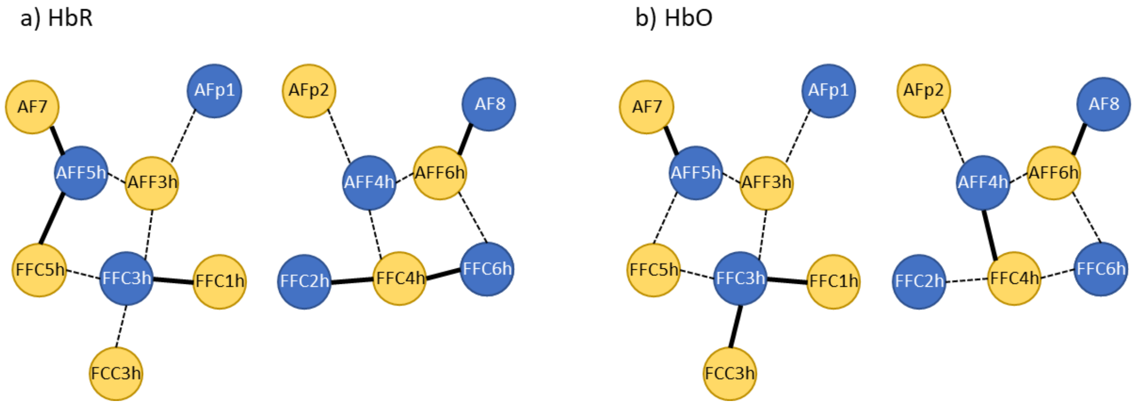

Figure S1. Channels with significant linear trends ($p < .05$, FDR-corrected[38]) in **a)** HbR and **b)** HbO. Blue = detector, yellow = source incl. short distance channel, dashed lines = fNIRS channel, bold lines = channel with significant linear trend.
